# Threshold Photoelectron Spectroscopy of the CH_2_I, CHI, and CI Radicals

**DOI:** 10.1021/acs.jpca.1c03874

**Published:** 2021-07-07

**Authors:** David V. Chicharro, Helgi Rafn Hrodmarsson, Aymen Bouallagui, Alexandre Zanchet, Jean-Christophe Loison, Gustavo A. García, Alberto García-Vela, Luis Bañares, Sonia Marggi Poullain

**Affiliations:** † Departamento de Química Física (Unidad Asociada I+D+i al CSIC), Facultad de Ciencias Químicas, Universidad Complutense de Madrid, 28040 Madrid, Spain; ‡ Synchrotron SOLEIL, L’Orme des Merisiers, St. Aubin, BP 48, 91192 Gif sur Yvette, France; ¶ Instituto de Física Fundamental, Consejo Superior de Investigaciones Científicas, C/Serrano, 123, 28006 Madrid, Spain; § Laboratoire de Spectroscopie Atomique, Moléculaire et Applications-LSAMA LR01ES09, Faculté des Sciences de Tunis, Université de Tunis El Manar, 2092 Tunis, Tunisia; ∥ ISM, Université Bordeaux 1, CNRS, 351 cours de la Libération, 33405 Talence Cedex, France; ⊥ Instituto Madrileño de Estudios Avanzados en Nanociencia (IMDEA-Nanoscience), Cantoblanco, 28049 Madrid, Spain

## Abstract

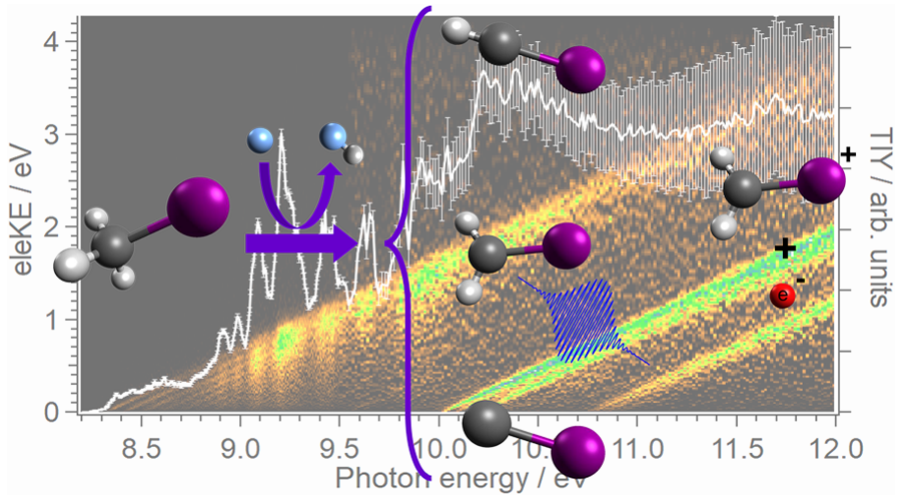

VUV photoionization of the CH_*n*_I radicals (with *n* = 0, 1, and 2) is investigated by means of synchrotron radiation coupled with a double imaging photoion-photoelectron coincidence spectrometer. Photoionization efficiencies and threshold photoelectron spectra (TPES) for photon energies ranging between 9.2 and 12.0 eV are reported. An adiabatic ionization energy (AIE) of 8.334 ± 0.005 eV is obtained for CH_2_I, which is in good agreement with previous results [8.333 ± 0.015 eV, SztárayJ. Chem. Phys.2017, 147, 0139442868839110.1063/1.4984304], while for CI an AIE of 8.374 ± 0.005 eV is measured for the first time and a value of ∼8.8 eV is estimated for CHI. *Ab initio* calculations have been carried out for the ground state of the CH_2_I radical and for the ground state and excited states of the radical cation CH_2_I^+^, including potential energy curves along the C–I coordinate. Franck–Condon factors are calculated for transitions from the CH_2_I(X̃^2^B_1_) ground state of the neutral radical to the ground state and excited states of the radical cation. The TPES measured for the CH_2_I radical shows several structures that correspond to the photoionization into excited states of the radical cation and are fully assigned on the basis of the calculations. The TPES obtained for the CHI is characterized by a broad structure peaking at 9.335 eV, which could be due to the photoionization from both the singlet and the triplet states and into one or more electronic states of the cation. A vibrational progression is clearly observed in the TPES for the CI radical and a frequency for the C–I stretching mode of 760 ± 60 cm^–1^ characterizing the CI^+^ electronic ground state has been extracted.

## I Introduction

The photochemistry of radicals and reactive intermediates is particularly relevant for atmospheric chemistry and interstellar science.^1^ Halocarbocations, as reaction intermediates, play an important role in gas-phase reactions, e.g., with ozone producing carbon monoxide and can undergo multiple reactions such as the formation of adducts with N- and O-containing molecules or the functionalization of aromatic molecules.^2,3^ The photodynamics of radicals and reactive intermediates such as halocarbocations have not received much attention due to the limited sources to produce such species. A radical source consisting of a microwave-discharge flow tube coupled with a double-imaging photoion photoelectron coincidence (i^2^PEPICO) spectrometer employing VUV tunable light was developed and installed at the *Dichroïsme Et Spectroscopie par Interaction avec le Rayonnement Synchrotron* (DESIRS) beamline at SOLEIL synchrotron.^4^ This setup has been successfully employed to study the photoionization of several radicals and reaction intermediates of atmospheric and interstellar interest, such as alkyl, methoxy, and methyl peroxy radicals.^5−12^ Here, this setup is used to study the photoionization of three iodized radicals, CH_2_I, CHI, and CI, produced by hydrogen abstraction reactions of atomic fluorine with methyl iodide. These species are part of the rich atmospheric photochemistry of iodine, which is particularly relevant in the oxidizing power of the atmosphere as well as in the formation of ultrafine aerosol particles.^13,14^

Although the electronic structure of the iodomethyl radical (CH_2_I) and its photodynamics have been scarcely investigated, its ground state has been thoroughly characterized both experimentally and theoretically.^15−19^ This radical of planar—or quasi-planar—equilibrium geometry is characterized by an enhanced C–I bond strength through π-bonding. The vibrational frequencies for several normal modes in the electronic ground state have been determined using different techniques, including infrared (IR) spectroscopy. In particular, Smith and Andrews^15^ assigned a frequency of ν_3_ = 611.2 cm^–1^ to the C–I stretching mode, which was also confirmed later by Baughcum and Leone.^16^ More recently, Bailleux et al.^19^ reported a detailed hyperfine microwave spectroscopy study where an equilibrium distance *R*_CI_ of 2.0388 Å was found. This value is in agreement with the calculations published by Odelius et al.^17^ and by Schwartz and co-workers^18^ where *R*_CI_ of 2.066 and 2.049 Å were obtained, respectively.

In a photoion–photoelectron coincidence experiment, Lin and co-workers detected for the first time the CH_2_I^+^ cation arising from the dissociation of CH_2_I_2_^+^ by one-photon ionization.^20^ The first photoelectron spectrum of CH_2_I produced by the reaction of CH_2_I_2_ with atomic fluorine was measured later by Andrews et al.^21^ On the basis of this spectrum, which is characterized by a single unstructured signal, vertical ionization and adiabatic ionization energies of 8.52 ± 0.03 and 8.40 ± 0.03 eV were determined, respectively. More recently, Sztáray et al.^22^ measured the CH_2_I photoelectron spectrum selected in mass and a vibrational progression was clearly observed in contrast to Andrews et al.^21^ A vibrational frequency of 710 cm^–1^ was found and assigned to the C–I stretching mode (ν_3_) while the adiabatic ionization energy was revised to 8.333 ± 0.015 eV. The authors suggested that the relative intensity and broadening of the peaks was due to less favorable transitions to other vibrational modes, in particular a H–C–H neutral wagging mode and some combination bands. The vibrational and electronic structure of the CH_2_I^+^ cation was investigated by Tao et al.^23^ on the basis of fluorescence excitation and emission spectroscopy. The CH_2_I^+^ cation in its X̃^1^A_1_ ground state, produced by a pulsed electrical discharge of the precursor CH_2_I_2_, was excited through a one-photon transition in the visible into an excited electronic state of A_1_ symmetry, lying ∼1.88 eV above. The C–I stretching vibrational mode was found to be characterized by a 746 cm^–1^ frequency in the cation ground state, slightly larger than the one measured by Sztáray et al.^22^ and considerably higher compared to the neutral ground state of the radical (611.2 cm^–1^),^15,16^ and in general to other halomethanes containing a single C–I bond, e.g., in CH_3_I, 533 cm^–1^. A similar effect was reported in a theoretical study in the bromomethyl radical CH_2_Br, where the vibrational frequency for the C–Br stretching mode appeared to be 160 cm^–1^ higher in the cation ground state than in the neutral one. The C–I stretching vibrational frequency (571.3 cm^–1^) in the excited electronic state of CH_2_I^+^ is also considerably lower than in the ground state. The large ν_3_ characterizing the CH_2_I^+^ (X̃^1^A_1_) is consistent with a resonance delocalization of the positive charge: H_2_C^+^—I ↔ H_2_C=I^+^, leading to a C—I partial double bond.

The photodynamics of CHI and CI have been scarcely investigated and are even less known than the iodomethyl radical. CHI, the simplest and most elusive iodocarbene, was first observed only in 2008 by Tao et al.^24^ using fluorescence excitation and emission spectroscopy. Carbenes are important reactive intermediates, characterized by a divalent carbon atom. Most studies focus on the determination of the multiplicity (singlet or triplet) characterizing their respective ground state and of the energy splitting—or gap—between the low-lying singlet and triplet states.^25−27^ After some discrepancies, the CHI ground state was established to be of singlet multiplicity (X̃^1^A′), while the triplet electronic state, ã^3^A″, lies only ∼0.17 eV above. Tao et al.^28^ also reported a detailed spectroscopic study on the X̃^1^A′ → Ã^1^A″ transition, measuring the vibrational frequencies, in both electronic states, in particular in the C–I stretching mode. Regarding the iodocarbyne (CI radical), a few studies, mainly theoretical, have been published on its electronic structure, in particular on the ground electronic state (X^2^Π) and on a manifold of electronic excited states correlating to the first two dissociation limits.^26,29−32^ The potential energy curves characterizing the ground and first excited states of the cation CI^+^ were recently calculated and adiabatic ionization energies of 8.287 and 8.347 eV were obtained depending on the level of theory.^33^ To the best of our knowledge, this is the only report regarding the cation CI^+^ structure and the ionization energies. The cations CHI^+^ and CI^+^ have not been further considered and the adiabatic ionization energies have not been experimentally determined.

In the present work, the photoionization of the radicals CH_2_I, CHI, and CI is investigated by i^2^PEPICO in combination with synchrotron radiation. For each molecule, the threshold photoelectron spectrum along with the photoionization yield curve allow us to determine the respective ionization energies and give information on the electronic structure of the cation, in particular for CH_2_I^+^. Potential energy curves of the first nine spin–orbit electronic states of the CH_2_I^+^ radical cation along with the ground state of the neutral radical have been computed and the corresponding Franck–Condon factors reflecting vertical photoionization from the CH_2_I (X̃^2^B_1_) in its vibrational ground state have been obtained. The experimental and theoretical methodologies are described in Section II, while the results are presented and discussed in Section III.

## II Methods

### II.1 Experiment

Experiments have been performed at the DESIRS beamline of the French synchrotron SOLEIL,^34^ on the permanent end-station SAPHIRS.^35^ The continuous microwave discharge flow-tube reactor used in the present experiments is composed of a 1 in. internal diameter quartz reactor and a movable quartz injector that slides inside the reactor.^4^ The distance between the injector and the first skimmer—placed at the end of the reactor—defines the reaction time, which can be adjusted within a range of a few milliseconds. A 20 sccm (standard cubic centimeters per minute) flow of commercial methyl iodide (CH_3_I) from Sigma-Aldrich is seeded in a 100 sccm flux of pure helium. The resulting mixture is fed into the flow-tube reactor through an injector. A flow of 40 sccm of a 5% mixture of F_2_/Ar is diluted with 1000 sccm of pure He and traversed a continuous 2.5 GHz microwave (MW) discharge, where 100% of the F_2_ is converted into F atoms before entering the reactor through a side arm. The total pressure in the reactor is kept at ∼0.94 mbar. The reaction time and concentrations are adjusted to maximize the signal of the three radicals produced by subsequent hydrogen abstractions: CH_2_I, CHI, and CI.
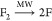
1
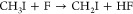
2
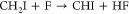
3
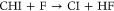
4

The output of the reactor traversed two skimmers before crossing the synchrotron VUV light at the center of the double imaging PEPICO (i^2^PEPICO) spectrometer DELICIOUS III.^36^ The momenta of the resulting photoelectrons and photoions are measured in coincidence. The mass resolving power *M*/Δ*M* is sufficient to separate CH_2_I (*m*/*z* = 140.9308), CHI (*m*/*z* = 139.9230), and CI (*m*/*z* = 138.9152). The tagged photoelectron images filtered in mass for each radical of interest are obtained as a function of the photon energy, and they are Abel inverted using the pBasex algorithm^37^ to provide the tagged electron signal as a function of electron kinetic energy and photon energy. The error bars shown throughout assume an initial Poisson distribution on the image pixel counts, propagated through all subsequent mathematical operations. The beamline was set to provide an estimated photon flux of 5 × 10^12^ photons/s, and steps of 5 meV for photon energies between 8.2 and 9.5 eV and of 15 meV between 9.5 and 12 eV. Spectral purity was ensured by means of a gas filter filled with Kr.^38^ A 200 L mm^–1^ grating was used and the monochromator slits were set to provide a photon energy resolution between 5 and 7 meV at photon energies ranging between 8.2 and 9.5 eV and a 3–4 meV resolution for photon energies varying between 9.5 and 12 eV. The well-known ionization energy of the methyl radical^39^ was employed to calibrate in situ the energy scale, along with the measured photoelectron spectrum from photoionization of FI,^40^ also produced in the reactor. The comparison between the absorption lines on the gas filter and the ionization energies on our PEPICO spectrometer has indeed demonstrated that all species are characterized by a similar Stark effect, a shift of *E*, where *E* is the electric field in V/cm.^41^ An accuracy on the energy scale of ±3 meV was obtained. The photon flux was recorded with a dedicated photodiode (AXUV, Optodiode) and used to normalize the data.

### II.2 Theory

To reproduce the measured photoelectron spectra, the one-dimension potentials associated with the C–I stretching mode of the CH_2_I and CH_2_I^+^ radicals have been calculated along the C–I internuclear distance *R*_CI_. To build this curve, which corresponds to a slice in the 6-D potential energy surface, the following procedure has been applied. For each value of *R*_CI_, the CH_2_ fragment was relaxed to minimize the energy of the cation ground state at CASPT2 level.^42^ For each relaxed geometry along the *R*_CI_ coordinate, state-average CASSCF^43^ calculations were performed considering an active space of 13 electrons in 11 orbitals (8 a′ and 3 a″ in *C*_*s*_ symmetry). To get a good description of the relative energy between the neutral and the cation, the ground state of the neutral species was included in the state average together with 10 singlet states (5 A′ and 5 A″) and 12 triplet states (6 A′ and 6A″) of the cation. Then, considering the resulting state-averaged optimized orbitals, the 22 electronic states of the cation and the ground state of the neutral species were considered at the multireference configuration interaction (MRCI) level.^44^ Finally, the spin–orbit matrix constructed with 12 triplet and 10 singlet states of the cation is calculated using the Breit–Pauli operator^45^ and all the states energetically accessible in the experiment are extracted from the matrix. All the calculations were done using the MOLPRO package^46^ considering the full electron ANO-RCC basis set.^47^ The Douglas–Kroll Hamiltonian was employed for a good description of the relativistic effects on the inner electrons of the iodine atom.

The resulting potential energy curves are then employed to compute the vibrational levels associated with the C–I stretch mode. The eigen-energies and eigen-functions of different vibrational states of the ground electronic state of CH_2_I and of the ground and excited electronic states of the CH_2_I^+^ cation have been calculated by solving the one-dimensional time-independent Schrödinger equation in the CH_2_–I (or (CH_2_–I)^+^) coordinate, using the *ab initio* MRCI potential energy curves for the electronic states. This is done by applying a Numerov–Cooley propagator in a finite grid of points in the above coordinate from 1.9 to 6.6 Å. Specifically, only the ground vibrational state *v* = 0 is calculated for the ground electronic state of CH_2_I and for all the excited electronic states of CH_2_I^+^, while several vibrational states from *v* = 0 to *v* = 10 have been obtained for the ground electronic state of CH_2_I^+^. Franck–Condon factors between the *v* = 0 vibrational state of the neutral CH_2_I system and all the calculated vibrational states of the different ground and excited electronic states of the cation are also obtained by computing the overlaps between the corresponding vibrational wave functions. The transition dipole moments coupling the ground electronic state of CH_2_I and all the electronic states of CH_2_I^+^ are assumed to be constant. We note that all the energies obtained have been arbitrarily shifted by 500 meV such that the ionization energy for the cation ground state matches the experimental adiabatic ionization energy (IE_ad_). This shift in energy is due to the low dimensionality of the present calculation.

## III Results and Discussion

**Figure 1:**
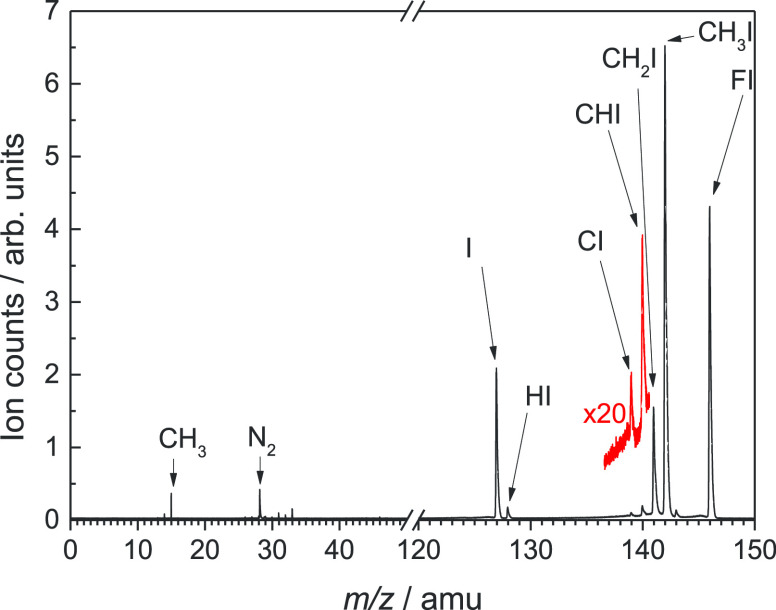
Time-of-flight mass spectrum (TOFMS) obtained by integrating all mass spectra for photon energies from 8.2 to 9.5 eV. The red line shows an expanded view around *m*/*z* 139 and 140.

The measured time-of-flight mass spectrum (TOFMS) accumulated over photon energies ranging between 8.2 and 9.5 eV is depicted in Figure 1. While the major peak observed at *m*/*z* ∼ 142 corresponds to the precursor, methyl iodide, three nearby signals are observed at *m*/*z* 141, 140, and 139 reflecting the formation of the three species of interest, CH_2_I, CHI, and CI, respectively. As observed, the peak intensity for these three species is considerably lower than the one of the precursor and decreases notably as a function of the number of hydrogen abstractions (see eqs 1–4) needed to produce each radical. Additional peaks at channel mass *m*/*z* 15, 127, 128, and 146 are attributed to CH_3_, I, HI, and FI, produced in secondary reactions in the reactor. In addition, some signal at *m*/*z* 28 due to the residual N_2_ present in the chamber is observed along with a small peak at *m*/*z* 143 from ^13^CH_3_I.

**Figure 2:**
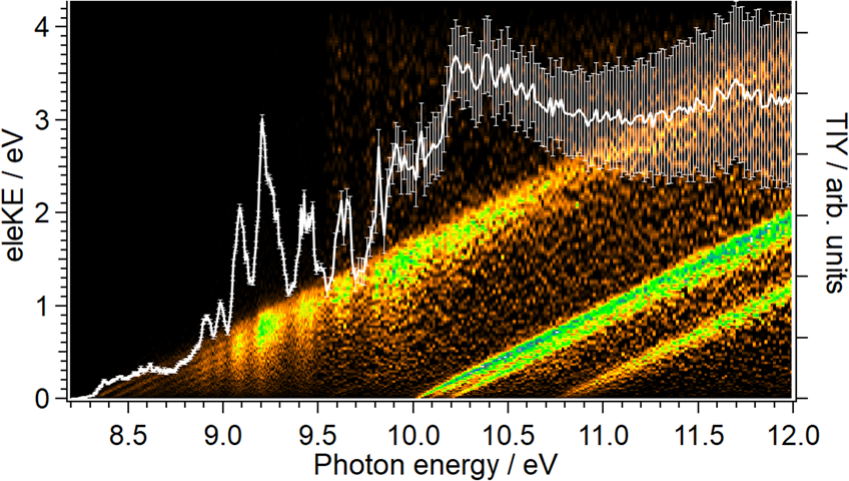
Intensity colormap representing the electron signal as a function of electron kinetic energy (eleKE) and photon energy for the *m*/*z* 141 channel (CH_2_I). The white curve with error bars represents the photoionization yield (PIY) as a function of photon energy, in terms of total ion yield (TIY).

Figure 2 shows the electron signal as a function of the electron kinetic energy and photon energy for the *m*/*z* 141 channel (CH_2_I). Due to energy conservation, several diagonal lines of constant unity slope, known as constant ionic state (CIS) lines are observed arising from each populated cationic state *i*. The corresponding slope, equal to 1, is represented by eleKE/(*hν* – *E*_*i*_), where eleKE is the electron kinetic energy, IE_*i*_ is the ionization energy of the *i*th state, and *hν* is the photon energy. Four main CIS lines are observed in Figure 2 reflecting the photoionization into at least the first four cationic states of CH_2_I.

The projection of the matrix in Figure 2 over the abscissa axis, known as the photoionization yield (PIY) curve, is obtained by integrating over all electron energies and is also plotted in Figure 2 as a white line with error bars. The onset observed at around 8.3 eV reflects the ionization energy (IE), to produce CH_2_I^+^ in its ground state. Different structures are in addition observed between 8.9 and 9.8 eV, attributed to autoionizing Rydberg states of the neutral radical CH_2_I.

The photoelectron signal matrix, as in Figure 2, contains a wealth of spectroscopic information, including the threshold photoelectron spectrum (TPES), which unveils the spectroscopic fingerprints of the cation. The TPES is obtained by integrating the signal along the CIS lines over only the slowest photoelectrons,^48^ such as
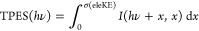
5where σ(eleKE) = 50 meV and *I*(*h*ν,eleKE) is the coincident signal intensity as a function of the photon and electron energies, as depicted in Figure 2. In the resulting TPES, only transitions between neutral and cationic states that are resonant with the photon energy will appear.

**Figure 3:**
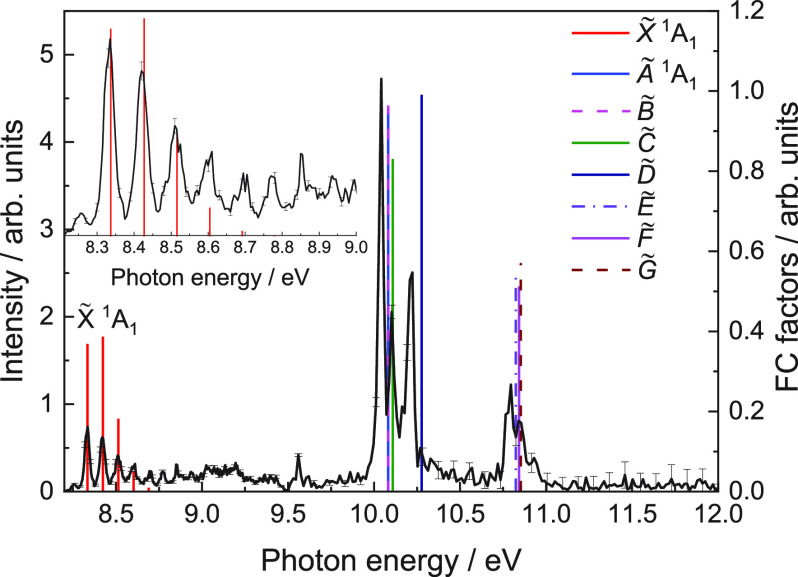
Threshold photoelectron spectrum (TPES) for the *m*/*z* 141 channel (CH_2_I), derived from signal integration for electrons with maximum kinetic energy of 50 meV (see Figure 2).

The TPES obtained for the *m*/*z* 141 channel (CH_2_I) is represented in Figure 3. A first vibrational progression lying at low photon energies up to ∼9 eV is clearly resolved and assigned to photoionization into CH_2_I^+^ in its electronic ground state. This vibrational structure, in great agreement with previous experiments reported by Sztaray et al.,^22^ is characterized by a separation between peaks (*Δν*) of 92.2 meV, which is consistent with the C–I stretching mode frequency of 746 cm^–1^ reported by Tao et al.^23^ While a first small peak, lying at ∼8.2 eV, is assigned to a hot band, the next peak, the first one of vibrational progression can be attributed to the vibrational ground state of CH_2_I^+^ (X̃^1^A_1_) and provides an experimental value for the adiabatic ionization energy IE_ad_ of 8.334 ± 0.005 eV. This value is consistent with previous determinations by Andrews et al.^21^ and Sztáray et al.,^22^ who reported values of 8.40 ± 0.03 and 8.333 ± 0.015 eV, respectively. Three additional peaks are observed lying at 10.04, 10.10, and 10.22 eV, while a bimodal structure appears at higher energies, peaking at 10.79 and 10.84 eV. In particular, the structure at 10.22 eV was previously assigned by Tao et al.^23^ to photoionization into the first excited state CH_2_I^+^ (Ã^1^A_1_).

**Figure 4:**
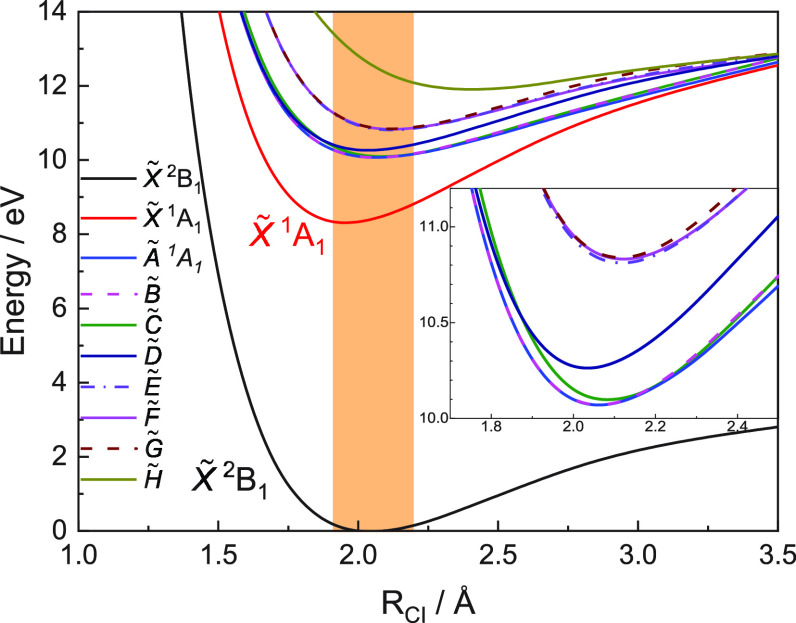
Computed potential energy curves as a function of C–I distance for the first nine electronic states of CH_2_I^+^ and the ground electronic state of CH_2_I. The orange rectangle illustrates the Franck–Condon region associated with the CH_2_I radical in its ground state. An expanded view of the potential energy curves for the excited electronic states of CH_2_I^+^ is shown in the inset.

The potential energy curves computed as a function of C–I distance for the first ten electronic states of CH_2_I^+^ and for the ground state of CH_2_I are depicted in Figure 4, while the corresponding ionization energy for each cationic state along with the computed zero point energy (ZPE) and the Franck–Condon factors from neutral CH_2_I in its vibrational ground state to the vibrational ground state of the C–I stretching mode of the first eight electronic states of the CH_2_I^+^ radical cation are summarized in Table 1. Table 2 shows the calculated FC factors for the 0–*v* vibronic transitions of the C–I stretching mode for the CH_2_I (X̃^2^B_1_) → CH_2_I^+^ (X̃^1^A_1_) transition. A high density of electronic states is observed in this somewhat small energy region (8–12 eV). The cation ground state, X̃^1^A_1_, lying at 8.33 eV, presents a pronounced bound shape, while the next four excited states, also bound, lie between ∼10.0 and ∼10.2 eV and the next three states almost overlap around 10.8 eV. The last excited electronic state labeled H̃ is loosely bound and appears at higher energies around 12.5 eV. As observed in Figure 4, all the cationic electronic states considered in the present calculations lead to the first dissociation limit, [CH_2_ + I]^+^, in their respective electronic ground states. At the moment, we cannot determine the charged fragment at the dissociation limit since methylene and atomic iodine have a particularly similar ionization energy of 10.396 and 10.451 eV, respectively.^49^ Further calculations on the dissociation of CH_2_I are in progress. The equilibrium internuclear distance *R*_CI_ characterizing the cation, in particular, the CH_2_I^+^ (X̃^1^A_1_) ground state, is slightly smaller than that of the neutral ground state CH_2_I (X̃^2^B_1_). This geometry change increases considerably the overlap between the vibrational wave functions between the initial neutral ground state and C–I stretch vibrational states of the ground state of the cation as reflected in the FC factors shown in Table 2.

**Table 1:** Computed Zero Point Energy (ZPE), Ionization Energy (IE), and Franck–Condon (FC) Factors for the 0–0 Vibronic Transitions of the C–I Stretching Mode for the Eight First Electronic States of CH_2_I^+^ with Respect to the Ground State CH_2_I(X̃^2^B_1_)^a^

species	state	ZPE (eV)	IE (eV)	FC factor	experiment
CH_2_I	X̃^2^B_1_	0.0382			
CH_2_I^+^	X̃^1^A_1_	0.0454	8.334	0.369	8.334
	Ã	0.0330	10.081	0.957	10.04
	B̃	0.0335	10.083	0.962	
	C̃	0.0323	10.109	0.831	10.10
	D̃	0.0356	10.277	0.991	10.22
	Ẽ	0.0341	10.824	0.540	10.79
	F̃	0.0329	10.843	0.513	10.84
	G̃	0.0346	10.853	0.568	

a Experimental values corresponding to the positions of some peaks in the TPES (see Figure 3) are specified for comparison.

**Table 2:** Calculated Franck–Condon (FC) Factors for the 0–*v* Vibronic Transitions of the C–I Stretching Mode for the Photoionization from the CH_2_I(X̃^2^B_1_) Electronic Ground State to the CH_2_I^+^(X̃ ^1^A_1_) Cationic Ground State

*v* state	FC factor
0	0.369
1	0.387
2	0.182
3	0.051
4	0.010
5	0.001
6	1.2 × 10^–4^
7	1.0 × 10^–5^

Vertical bars representing the calculated Franck–Condon factors associated with photoionization into the ground and first excited electronic states of CH_2_I^+^ (see Table 1) are depicted in Figure 3. A general good agreement is found although some discrepancies on the relative intensities are observed. These can be directly related to the reduced dimensionality of the calculations. The vibrational progression between 8.3 and 8.8 eV reflecting photoionization into the X̃^1^A_1_ cationic ground state vibrationally excited in the C–I stretching mode is well reproduced. A frequency of 90.2 meV, i.e., 727.5 cm^–1^, is obtained from the calculation, which is in good agreement with the frequency of 92 meV, i.e., 742 cm^–1^, extracted from the measured TPES depicted in Figure 3. This last value is consistent with that reported by Tao et al.^23^ of 746 cm^–1^. The peak observed in Figure 3 at 10.042 eV can be attributed to photoionization into the first Ã and second B̃ electronic excited states of the cation, which are not degenerate but they almost overlap. The structure at 10.102 eV is similarly assigned to photoionization into the C̃ state, while the peak at 10.222 eV corresponds to photoionization into the D̃ excited state. This last electronic state was previously misassigned to the Ã state by Tao et al.^23^ in their spectroscopic study. Finally, the formation of CH_2_I^+^ in either Ẽ, F̃, or G̃ states could be responsible for the structure arising at ∼10.8 eV.

The good agreement obtained between theory and experiment shows that the MRCI approach is appropriate to accurately describe ionization energies and electronic excited states. It also emphasizes the importance to account accurately for the spin orbit term when iodine is present. The spin–orbit coupling between singlet and triplet states can indeed reach up to ∼2900 cm^–1^, which is of the order of the splitting between electronic states.

**Figure 5:**
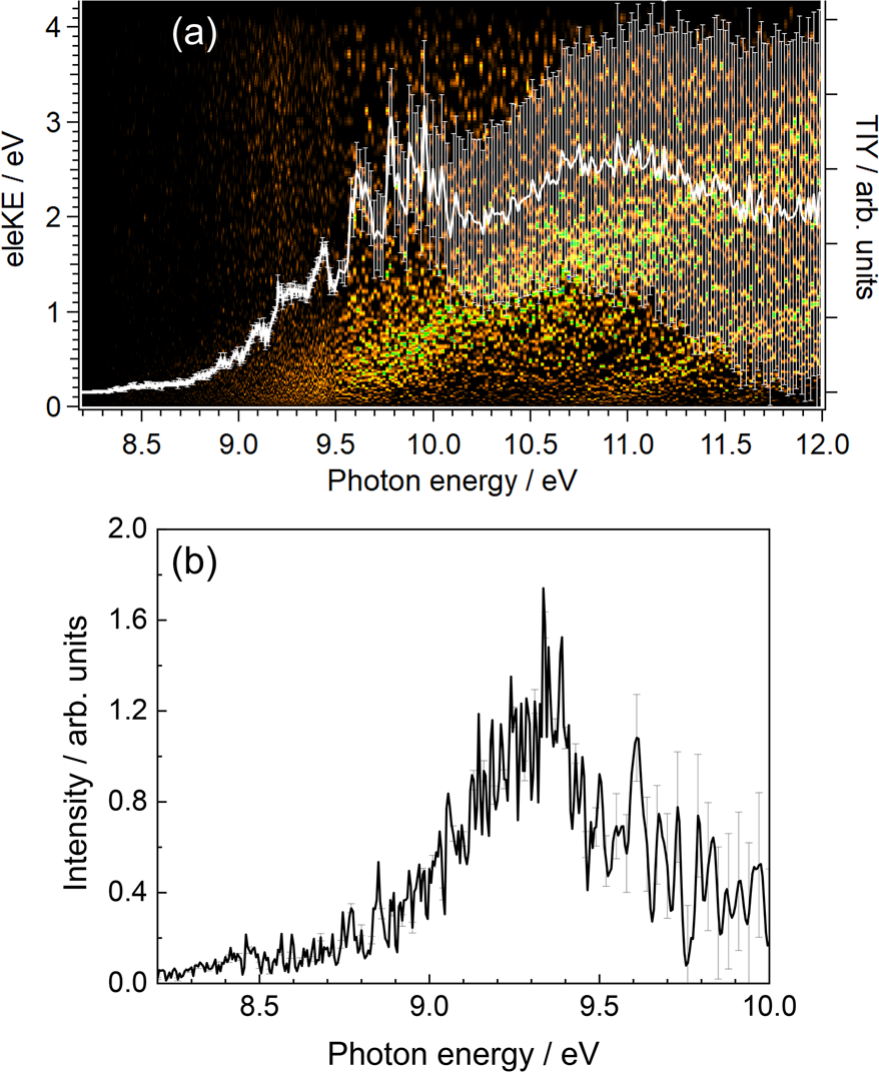
(a) Intensity colormap representing the electron signal as a function of electron kinetic energy (eleKE) and photon energy for the *m*/*z* 140 channel (CHI). The white curve with error bars corresponds to the photoionization yield (PIY) as a function of photon energy. (b) Threshold photoelectron spectrum (TPES) for CHI derived from signal integration of electrons with a maximum kinetic energy of 50 meV. Only photon energies below 9.5 eV are plotted due to the low signal-to-noise ratio above.

**Figure 6:**
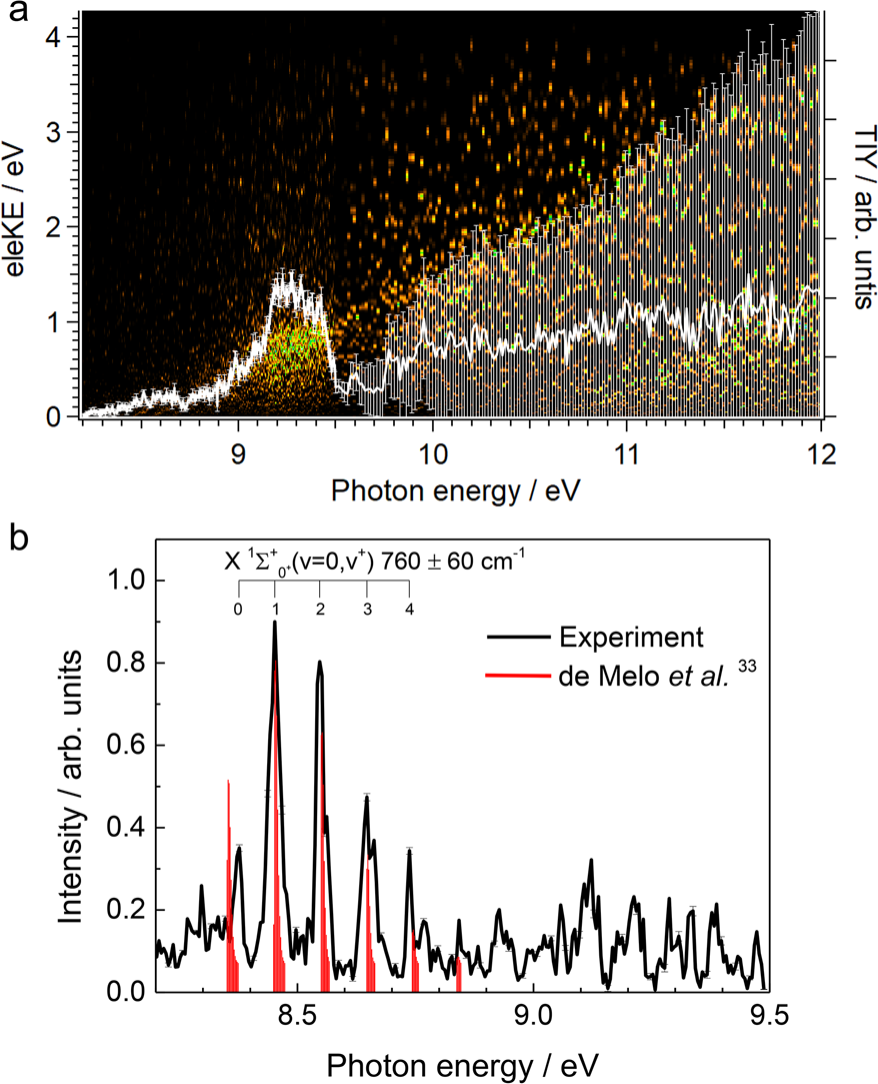
(a) Intensity colormap representing the electron signal as a function of electron kinetic energy (eleKE) and photon energy for the *m*/*z* = 139 channel (CI). The white curve with error bars corresponds to the photoionization yield (PIY) as a function of photon energy. (b) Threshold photoelectron spectrum for CI, derived from the signal integration for electrons with a maximum kinetic energy of 50 meV. Only photon energies below 9.5 eV are plotted due to the low signal-to-noise ratio above this value. The red sticks have been taken from the 300 K simulation reported by de Melo et al.^33^ and shifted by +6 meV with respect to their calculated ionization energy of 8.347 eV obtained at the RCCSD(T) level.

The electron signal as a function of the electron kinetic and photon energies along with the PIY curve for *m*/*z* 140 and 139, i.e., CHI and CI species, are depicted in Figures 5 and 6, respectively. As observed in the PIY curves, the error bars are significant for photon energies higher than 9.5 eV. The signal is considerably low at these energies, and the signal-to-noise ratio is close to one. Therefore, we only consider data for photon energies below 9.5 eV.

Considering first the CHI carbene, the PIY rises at around ∼8.8 eV and three shoulders are observed between 9.0 and 9.5 eV that might reflect some autoionizing states although they cannot be properly assigned due to the low signal. The TPES for CHI is similarly obtained from Figure 5a by using eq 5 and is depicted in Figure 5b for photon energies below 9.5 eV. Due to the weak Franck–Condon factors for transitions to the ground cationic state, we can only report an observed ionization energy of ∼8.8 eV based on Figure 5b, with a vertical ionization energy at 9.34 eV since it presents a single broad structure peaking at that energy. This peak could reflect photoionization from both the singlet and triplet electronic states, X̃^1^A′ and a^3^A″, which are expected to be close in energy,^24^ and therefore both present in the flowtube. Some geometry distortion between the neutral and the cation ground states, leading to favored photoionization into several vibrational levels of the cation ground state, could also explain the broad structure observed. *Ab initio* calculations of the CHI^+^ cation are in progress to disentangle the contribution to the photoionization of the singlet and triplet states of the neutral and the possible effect of the geometry.

As observed in Figure 6a, a small increase from ∼8.3 eV and a main broad peak for photon energies between 9.0 and 9.5 eV characterize the CI radical. The corresponding TPES, depicted in Figure 6b, shows a marked vibrational progression. de Melo et al.^33^ recently reported a theoretical study of the direct ionization of CI. They calculated adiabatic energies of 8.287 and 8.347 eV at the CASSCF/MRCI and RCCSD(T) levels of theory, respectively. They also performed a Franck–Condon simulation for transitions to the X^1^Σ^+^ ground cationic state and observed a vibrational progression reflecting the shortening of the bond distance from neutral (2.037 Å^31^) to cation (1.926 Å^33^). However, the imperfect agreement between simulated and experimental spectra, coupled to the low signal-to-background and the potential presence of autoionizations affecting the relative branch ratios, makes assignment of the adiabatic value challenging. Indeed, calculations place the value of the vertical transition at (v, v^+^) = (0, 1), and using the experimental vertical transition (the most intense band at 8.45 eV) as a reference to match the simulated spectrum (see Figure 6b) yields a very good agreement for the high energy part between 8.4 and 8.8 eV. From the bands within this interval, we obtain an experimental vibrational frequency of 760 ± 60 cm^–1^ for the ground state of CI^+^, in agreement with the calculated value of 810 cm^–1^ (ref (33)) within our error bars and noticeably larger than the value of 630 cm^–1^ calculated for the neutral X^1^Σ^+^ ground state.^31^ This difference in the C–I stretching frequency for the cation and neutral ground state was also observed in the CH_2_I radical, as discussed previously, and in other diatomic species such as IO, where the vibrational frequency goes from 682 cm^–1^ in the neutral ground state to 810 cm^–1^ in the cation ground state, as the I–O bond length shortens.^50^ The agreement, however, is less satisfactory regarding the experimental band at 8.37 eV corresponding to the adiabatic transition, which is shifted by 190 cm^–1^ to the blue with respect to the predicted position. Interestingly, this shift is very close to the calculated frequency differences between the neutral (630 cm^–1^ in the X^1^Σ^+^, ref (31)) and the cation (810 cm^–1^, ref (33)), which means that it could conceivably be assigned to a hot band (1, 0). We note that the appearance of hot bands is most often the case in species produced by H abstraction due to the large exothermicity for formation of HF. Therefore, although the assignment shown in Figure 6b leads to an experimental adiabatic ionization energy of 8.374 ± 0.005 eV, in very good agreement with that calculated at the RCCSD (T) level by de Melo et al.,^33^ an alternative assignment could place the adiabatic value at 8.452 eV if the first band is considered as a hot band.

## IV Conclusions

The photoionization of CH_*n*_I species (with *n* = 0, 1, and 2) is investigated by means of synchrotron radiation at the DESIRS beamline in conjunction with a photoion–photoelectron coincidence spectrometer (DELICIOUSIII) at the SOLEIL synchrotron, France. Radicals CH_2_I, CHI, and CI are produced in a microwave-discharge flow tube, by hydrogen abstraction reaction of fluorine with methyl iodide. Photoionization yield (PIY) curves and threshold photoelectron spectra (TPES) are reported for the three species as a function of the photon energy in the 9.2–12.0 eV range. *Ab initio* calculations have been carried out for the CH_2_I radical, including potential energy curves along the C–I coordinate and the Franck–Condon factors for vibronic transitions from the CH_2_I(X̃^2^B_1_) ground state of the neutral radical. The TPES for CH_2_I is characterized by a first vibrational progression associated with the photoionization into CH_2_I^+^ ground state vibrationally excited in the C–I stretching mode and by several structures at higher energies corresponding to the photoionization into excited cationic states. All these structures are assigned on the basis of the calculated Franck–Condon factors. An adiabatic ionization energy of 8.334 ± 0.005 eV is obtained for CH_2_I, in good agreement with previous work. Although a considerably lower signal characterizes the results for CHI and CI, their adiabatic ionization energy, determined for the first time in this work, are ∼8.8 and 8.374 ± 0.005 eV, respectively. The TPES obtained for the CHI is characterized by a broad structure peaking at 9.335 eV, which could be due to the photoionization from both singlet and triplet states and into one or more electronic states of the cation. A vibrational progression is clearly observed in the TPES of the CI radical and a frequency for the C–I stretching mode of 760 ± 60 cm^–1^ for the CI^+^ electronic ground state has been determined.
